# [Retracted] FAM172A modulates apoptosis and proliferation of colon cancer cells via STAT1 binding to its promoter

**DOI:** 10.3892/or.2023.8545

**Published:** 2023-04-13

**Authors:** Kai Qian, Jinqian Zhang, Jingbo Lu, Wenjun Liu, Xingxing Yao, Qing Chen, Shun Lu, Guoan Xiang, Hao Liu

Oncol Rep 35: 1273–1280, 2016; DOI: 10.3892/or.2015.4485

Following the publication of this paper, it was drawn to the Editor's attention by a concerned reader that lanes 1–3 of the EMSA results shown in Fig. 6 on p. 1278 were strikingly similar to data that had already appeared in a different form in the following publication by different authors at different research institutes: Qiu K, Li Z, Chen J, Wu S, Zhu X, Gao S, Gao J, Ren G and Zhou X: EIN3 and ORE1 accelerate degreening during ethylene-mediated leaf senescence by directly activating chlorophyll catabolic genes in *Arabidopsis*. PLoS Genet 11: e1005399, 2015.

Owing to the fact that the contentious data in the above article had already been published prior to its submission to *Oncology Reports*, the Editor has decided that this paper should be retracted from the Journal. After having been in contact with the authors, they accepted the decision to retract the paper. The Editor apologizes to the readership for any inconvenience caused.

